# Antagonisms of ASFV towards Host Defense Mechanisms: Knowledge Gaps in Viral Immune Evasion and Pathogenesis

**DOI:** 10.3390/v15020574

**Published:** 2023-02-19

**Authors:** Liangzheng Yu, Zhenbang Zhu, Junhua Deng, Kegong Tian, Xiangdong Li

**Affiliations:** 1 Jiangsu Co-Innovation Center for Prevention and Control of Important Animal Infectious Diseases and Zoonoses, College of Veterinary Medicine, Yangzhou University, Yangzhou 225009, China; 2 Joint International Research Laboratory of Agriculture and Agri-Product Safety, Ministry of Education, Yangzhou University, Yangzhou 225009, China; 3 Luoyang Putai Biotech Co., Ltd., Luoyang 471003, China; 4 College of Animal Science and Veterinary Medicine, Henan Agricultural University, Zhengzhou 450002, China

**Keywords:** African swine fever virus, immune evasion, pathogenesis

## Abstract

African swine fever (ASF) causes high morbidity and mortality of both domestic pigs and wild boars and severely impacts the swine industry worldwide. ASF virus (ASFV), the etiologic agent of ASF epidemics, mainly infects myeloid cells in swine mononuclear phagocyte system (MPS), including blood-circulating monocytes, tissue-resident macrophages, and dendritic cells (DCs). Since their significant roles in bridging host innate and adaptive immunity, these cells provide ASFV with favorable targets to manipulate and block their antiviral activities, leading to immune escape and immunosuppression. To date, vaccines are still being regarded as the most promising measure to prevent and control ASF outbreaks. However, ASF vaccine development is delayed and limited by existing knowledge gaps in viral immune evasion, pathogenesis, etc. Recent studies have revealed that ASFV can employ diverse strategies to interrupt the host defense mechanisms via abundant self-encoded proteins. Thus, this review mainly focuses on the antagonisms of ASFV-encoded proteins towards IFN-I production, IFN-induced antiviral response, NLRP3 inflammasome activation, and GSDMD-mediated pyroptosis. Additionally, we also make a brief discussion concerning the potential challenges in future development of ASF vaccine.

## 1. Introduction

African swine fever (ASF) was first discovered in Kenya in the 1920s and gradually spread across many regions covering the Caucasus, sub-Saharan Africa, and Eastern Europe [1]. The first outbreak of ASF in China was reported on 3 August 2018 [2,3]. Subsequently, the disease spread throughout the country with an unprecedented speed, causing huge economic losses to local pig industry [4]. Later, the emergence and coexistence of naturally mutated, low-virulent genotype I and II ASFV field strains has posed more challenges for this disease’s prevention and control [5,6]. 

African swine fever virus (ASFV) is the causative agent of ASF. It is an enveloped, icosahedral, double-stranded DNA arbovirus with a genomic length ranging from 170 to 193 kilobase (kb) and is the only member of *Asfarviridae* family [7,8,9]. ASFV exhibits complexity because of its large genome since this virus can encode multifunctional proteins enough for its productive replication [10]. Especially, it is most likely that ASFV possesses an entire transcription machinery of its own to synthesize viral mRNA, including DNA-dependent RNA polymerase subunit (e.g., pEP1242L, pNP1450L), transcription factor (e.g., pI243L), RNA helicase (e.g., pQP509L, pA859L), RNA capping enzyme (e.g., pNP868R), etc. [8]. Moreover, ASFV is known to encode DNA ligase (pNP419L), DNA polymerase X-like (pO174L), lambda-like exonuclease (pD345L), AP endonuclease (pE296R), and PCNA-like (pE301R), which are essential for the base excision repair (BER) [11]. The complicated genomic features make ASFV a difficult opponent.

Vaccines are recognized as the most useful tool to prevent and control viral infection [12,13]. Currently, advances have been made in the research of ASF live attenuated vaccines (LAVs). LAVs can confer some degree of protection against challenge with homologous parental strains, which is proven to be effective. Although these protective LAVs exhibit attenuated characteristics, their safety has always been a controversial issue. As reported, ASFV-G-ΔI177L, a candidate for ASF LAV, is constructed by deleting a single I177L gene from the highly virulent Georgia 2007 isolate. Such experimental vaccine candidates present as completely attenuated at a low or even high dose of inoculation. Vaccinated pigs all remain clinically normal, with low viremia titers and strong virus-specific antibody response [14]. However, assessment on the safety of vaccine candidate ASFV-G-ΔI177L shows that virus shedding from vaccinated pigs can be detected for couple of days [15]. Of interest, ASFV-G-ΔI177L was once approved for commercial use in June 2022 and two months later suspended for its safety issues by authorities in Vietnam [16]. Thus, caution should be maintained about the gene-deleted ASF LAVs.

Although other attempts for developing ASFV vaccines have also been conducted and evaluated in the past decades, no prophylactic and therapeutic vaccines are commercially available to effectively eradicate ASF epidemics [17,18]. ASFV adapts various strategies to suppress host immunity and escape from the innate and adaptive immune responses, which may be responsible for the restricted vaccine development [19,20,21]. For instance, previous studies reported that ASFV could disturb antigen processing and presentation targeting the expression of major histocompatibility complex (MHC) molecules [22]. Furthermore, several studies indicated that ASFV could also induce massive destruction of lymphocyte subsets, which is characterized by the apoptosis of bystander non-infected B and T lymphocytes in vivo [23,24].

To date, a few articles have already highlighted the negative impact of plentiful ASFV proteins on antiviral immune response [25,26,27]. However, several ASFV immunosuppressive proteins (e.g., pE184L, pD345L, pD117L) and novel immune evasion mechanisms have not been summarized. Thus, this review will focus on relevant studies that deeply elucidate the antagonisms of ASFV towards host defense mechanisms in the last five years (2019–2023). In the following sections, we aim to elaborate these mechanisms by which ASFV targets key signals to counteract host antiviral pathway in different manners, mainly analyze the implications that ASFV proteins-mediated antagonisms pose on viral replication, pathogenicity, and virulence in vivo, and briefly discuss some knowledge gaps that should be filled in the future research.

## 2. Suppression of IFN-I Production and IFN-Induced Antiviral Responses

**Figure 1:**
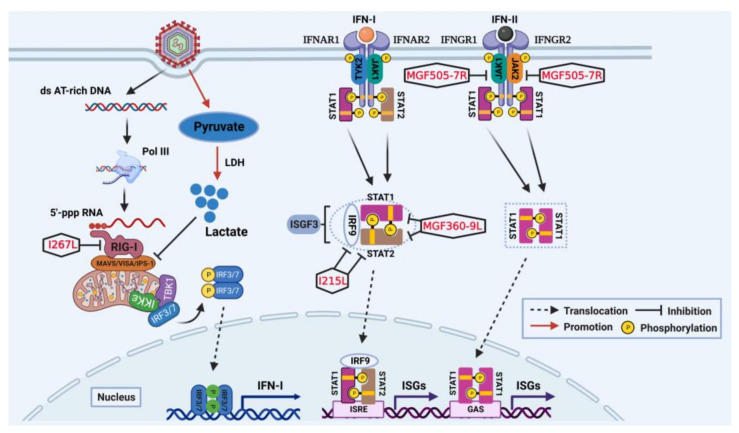
Schematic overview of impairment on IFN-I production targeting RIG-MAVS axis and IFN-induced antiviral responses by ASFV proteins in the latest reports. After ASFV infection, viral pI267L and increased lactate inhibit IFN-I production. Mechanistically, pI267L destroys the stabilized form of activated RIG-I and lactate to prevent the aggregation of MAVS. ASFV pI215L and pMGF360-9L inhibit IFN-I-induced antiviral response, while pMGF505-7R inhibit IFN-II-induced antiviral response. Mechanistically, pI267L mediates the degradation of IRF9 and STAT2, pMGF360-9L mediates the degradation of STAT1 and STAT2, and pMGF505-7R mediates the degradation of JAK1 and JAK2.

Type I interferon (IFN-I) serves as critical immune mediators for restricting the spread of viral infection [28,29]. Recent studies have showed that both cGAS-STING and RIG-I-MAVS pathways involve in the production of IFN-I during ASFV infection [30,31]. After secreted outside of cells, the IFN-I will bind their receptors on the cytomembrane and initiate the Janus kinase-signal transducer and activator of transcription (JAK-STAT) signaling cascade, leading to the transcriptional regulation of hundreds of IFN-stimulated genes (ISGs) [32,33,34]. This process presents a remarkable antiviral state in vivo [23]. Although IFN-I-induced immunity provides an effective line of defense, ASFV has evolved several strategies to antagonize it [35,36]. Indeed, ASFV encodes multiple proteins that can manipulate and evade host antiviral response by specific interactions with key elements of the JAK-STAT pathway, as illustrated in Figure 1. 

### 2.1. Impairment on IFN-I Production Targeting cGAS-STING Axis

Recent studies have demonstrated that several ASFV-encoded proteins targeting cGAS-STING pathway can inhibit IFN-I production in diverse manners (Table 1) [25]. In this pathway, cyclic GMP-AMP synthase (cGAS), which was previously identified to be a cytosolic DNA sensor, can directly recognize and bind to the non-self ASFV DNA [37]. After that, cGAS is activated and then catalyzes the synthesis of 2′3′-cyclic GMP-AMP (cGAMP) [37]. As a critical secondary messenger in the cGAS-STING pathway, cGAMP will bind to and activate stimulator of interferon genes (STING) [38]. However, cGAMP is likely to be cleaved by viral nucleases, which certainly restricts the STING-dependent IFN-I production [39,40]. Indeed, ASFV-encoded pEP364R and pC129R, homologs to the nuclease, exhibit strong phosphodiesterase activity [41]. Either pEP364R or pC129R can selectively interact with cGAMP, specifically mediating the cleavage of cGAMP with their enzyme activity [41].

ASFV is also likely to impair the IFN-I production by targeting critical proteins (e.g., STING, IRF3, TBK1) in the downstream cGAS-STING pathway. Firstly, activated STING will further recruit TANK-binding kinase 1 (TBK1) and IFN regulatory factor 3 (IRF3) to form a trimeric complex “STING-IRF3-TBK1” [42]. However, ASFV-encoded pE184L can prevent the formation of “STING-IRF3-TBK1” complex by impairing the oligomerization and dimerization of STING [43]. ASFV structural protein p17 can also disrupt the recruitment of TBK1 and Ikkε by interacting with STING as well although its implications on STING are not clearly elucidated [44]. Secondly, TBK1, a crucial kinase, is required for the IRF3 phosphorylation [45]. The kinase activity of TBK1 is strictly regulated by multiple post-translational modifications (PTMs), including ubiquitination and phosphorylation [46]. As reported, phosphorylation of serine residues at position 172 in the activation loop of TBK1 and K63-linked polyubiquitination of lysine residues at positions 30 and 401 of TBK1 can both activate its kinase activity [47,48]. Nevertheless, early-expressed ASFV pDP96R can significantly suppress the phosphorylation of TBK1 [49]. The only known viral ubiquitin-conjugating enzyme (UBCv1) encoded by the ASFV I215L gene can enhance RING finger protein (RNF)138 to degrade RNF128, which inhibits K63-linked polyubiquitination of TBK1 [50,51]. Except for the negative modulation on PTMs of TBK1, late-expressed pA137R can also mediate the autophagosome and lysosome-dependent degradation of TBK1 to block the STING-IRF3 signaling pathway [52]. Of note, ASFV-encoded pS273R, a member of the SUMO-1-specific protease family, can affect the SUMOylation of inhibitor of nuclear factor kappa-B kinase ε (Ikkε) by its catalytic activity [53]. Ikkε, a homolog of TBK1, seems to involve in the activation of STING-IRF3 signaling pathway although the mechanism is not fully clear [54]. Thirdly, phosphorylated IRF3 will dissociate from the complex and then translocate into the nucleus, which consequently triggers the transcription of interferon genes (e.g., IFN-I) [55]. Of note is that the transcriptional activity of IRF3 is also modulated by posttranslational modifications (e.g., phosphorylation). Nonetheless, ASFV pE301R, pI226R, and pE120R can suppress the phosphorylation of IRF3 to interfere with normal transcriptional function of IRF3 [56,57,58]. ASFV pM1249L, expressed in the late phase of ASFV infection, exhibits dual inhibitory effects on IFN-I production since pM1249L can not only suppress TBK1 phosphorylation but also mediate the lysosome-dependent degradation of IRF3 [59]. Taken together, interference with PTMs of critical signals is one of the major strategies employed by ASFV proteins to impair STING-mediated IFN-I production.

The multigene families (MGFs) of ASFV can exploit cellular protein degradation systems to impair STING-IRF3 signaling pathway mainly by mediating the degradation of critical signals. The MGFs locate at the left terminal 40 kb and right terminal 20 kb variable regions in ASFV genome [60]. They are mainly grouped into MGF-100, MGF-110, MGF-300, MGF-360, and MGF-505/530, whose gain or loss causes variation in the genomes of different ASFV isolates [61]. Among them, pMGF360-11L can mediate the caspase-, proteasome-, and autophagosome-dependent degradation of TBK1 and IRF7 [62]. pMGF505-11R can mediate the lysosomal-, proteasome-, and autophagosome-dependent degradation of STING [63]. The viral non-structural protein pMGF360-14L can mediate the degradation of IRF3 by facilitating E3 ligase TRIM21-mediated K63-linked ubiquitination [64]. Early-expressed pMGF505-7R executes multifaceted inhibition on STING-dependent antiviral responses [65]. pMGF505-7R can mediate proteasome-dependent degradation of TBK1, caspase-, autophagosome-, and proteasome-dependent degradation of IRF7 and autophagosome-dependent degradation of STING [66,67]. Additionally, pMGF-505-7R can also facilitate the degradation of STING by upregulating Unc-51-like autophagy-activating kinase 1 (ULK1) [67,68].

**Table 1:** Antagonisms of ASFV proteins in latest reports towards IFN-I production by targeting cGAS-STING axis.

ASFV Protein	Functional Site/Domain	Key Target	Underlying Mechanism	Reference
pEP364R, pC129R	Amino acids Y76 and N78 in pep364r	cGAMP	Selectively cleave cgamp	[41]
pE184L	Amino acids 1–20 in pe184l	STING	Impair STING oligomerization and dimerization	[43]
p17 (pD117L)	Amino acids 39–59 in p17	STING	Interfere with the recruitment of TBK1 and Ikkε	[44]
pDP96R	Amino acids 30–96 in pdp96r	TBK1	Suppress the phosphorylation of TBK1	[49]
pI215L (UBCv1)	Unknown	TBK1	Inhibit K63-linked polyubiquitination of TBK1	[50]
pA137R	Unknown	TBK1	Mediate the degradation of TBK1	[52]
pM1249L	Unknown	TBK1; IRF3	Suppress the phosphorylation of TBK1; mediate the degradation of IRF3	[59]
pE120R	Amino acids 72–73 in pe120r	IRF3	Suppress the phosphorylation of IRF3	[58]
pE301R	Amino acids 1–200 in pe301r	IRF3	Suppress the phosphorylation of IRF3	[56]
pI226R	Unknown	IRF3	Suppress the phosphorylation of IRF3	[57]
pS273R	Amino acids 1–20 and 256–273 in ps273r	Ikkε	Affect the sumoylation of Ikkε	[53]
pMGF360-11L	Amino acids 167–353 in pmgf360-11L	TBK1; IRF7	Mediate the degradation of TBK1 and IRF7	[62]
pMGF505-11R	Amino acids 1–191 and 182–360 in pmgf505-11R	STING	Mediate the degradation of STING	[63]
pMGF360-14L	Unknown	IRF3	Mediate the degradation of IRF3	[64]
pMGF-505-7R	Unknown	TBK1; IRF7	Mediate the degradation of TBK1 and IRF7	[66]
		STING	Mediate the degradation of STING	[67]

### 2.2. Impairment on IFN-I Production Targeting RIG-I-MAVS Axis

During infection, ASFV DNA, as a danger signal, will be released into the cytoplasm. Previous studies showed that DNA-dependent RNA polymerase III (Pol-III) can also detect cytosolic DNA and trigger the production of IFN-I through the RIG-I-MAVS pathway [69]. Pol-III serves AT-rich double-stranded DNA (dsDNA) as the template to transcribe it into double-stranded RNA (dsRNA) containing a 5′-triphosphate end [70]. The newly formed 5′-triphosphate RNA can be detected and tightly bound by RIG-I in the cytoplasm, which initiates the downstream signaling cascade [71]. K63-linked polyubiquitination has proven to be an important regulation for the RIG-I activation [72]. However, some viruses can effectively disturb the RIG-I-mediated antiviral signaling by targeting the process of ubiquitination [73,74]. Ran et al. confirmed that AT-rich regions of ASFV genomes can be recognized and transcribed into AU-rich 5′pppRNA transcripts by Pol-III [31]. They further demonstrated that ASFV virulence factor pI267L originating from either genotype I or II can potently antagonize the RNA Pol-III-RIG-I axis [31]. Mechanistically, pI267L destroys the stabilized and enhanced form of activated RIG-I by impairing Riplet-mediated K63-linked polyubiquitination, a critical step in RIG-I-MAVS signaling pathway [31,75,76].

Furthermore, viruses can exploit the host metabolism to synthesize plentiful metabolites required for their replication, which facilitates viral productive infection [77,78]. To be mentioned, the crosstalk between innate immunity and metabolism is well discussed in several reports [79,80,81]. Recent studies have revealed that ASFV can alter the host cellular metabolism to disrupt innate immune responses for self-replication by impairing RIG-I-mediate IFN-I production [82]. Mechanistically, ASFV infection triggers the increase of pyruvate production, giving rise to an enhanced level of lactate under the action of lactate dehydrogenase (LDH) [82]. Notably, lactate is a natural suppressor of RIG-I-mediated signaling pathway by targeting MAVS, which lowers the expression of beta interferon (IFN-β) [83]. However, the mechanisms by which ASFV can modulate cellular metabolism to promote productive infection have been rarely elucidated. More studies on the metabolomic analysis of ASFV-infected cells maybe provide novel insights into the association between metabolic regulation and immune evasion.

### 2.3. Impairment on IFN-Induced Antiviral Responses Targeting JAK-STAT Pathway

The activation of the JAK-STAT pathway by IFNs will upregulate the expression of hundreds of ISGs, leading to the remarkable restriction of viral spread and replication [84]. However, to survive and propagate within the host cells, ASFV has encoded multiple proteins to counteract the IFN-induced antiviral responses [36]. Recently, studies have suggested that ASFV proteins mainly block the JAK-STAT pathway by mediating the degradation of critical signals (e.g., JAKs, STATs, IRF9). The IFN-stimulated gene factor 3 (ISG3) complex is formed through the interaction of STAT1/STAT2 heterodimers with IRF9, which plays crucial roles in the IFN-I-triggered JAK-STAT pathway [32]. ASFV pI215L (UBCv1) can interfere with the formation of ISG3 in both ubiquitin-conjugating enzyme-activity-independent and dependent manners. ASFV pI215L (UBCv1) can interact with IRF9 and mediate the degradation of IRF9 via autophagy-lysosome pathway, which is independent of its ubiquitin-conjugating enzyme activity [85]. Additionally, ASFV pI215L (UBCv1) can also mediate the degradation of STAT2 via ubiquitin-proteasome pathway, which is dependent on its ubiquitin-conjugating enzyme activity [86]. In addition, Zhang et al. identified pMGF360-9L as also working as an inhibitor of JAK/STAT pathway [87]. Mechanically, pMGF360-9L inhibits IFN-β-induced ISGs transcription by mediating the degradation of STAT1 and STAT2 through the apoptotic pathway and ubiquitin-proteasome pathway, respectively [87]. Li et al. reported that pMGF-505-7R inhibited the IFN-γ-mediated JAK-STAT axis [88]. Mechanistically, pMGF-505-7R is found to interact with JAK1 and JAK2 and mediates their degradation by upregulating E3 ubiquitin ligase RNF125 expression and inhibiting expression of Hes5, respectively [88].

## 3. Inhibition of NLRP3 Inflammasome Activation and GSDMD-Mediated Pyroptosis

The NACHT, LRR, and PYD domains-containing protein 3 (NLRP3) inflammasome has become an indispensable component of host innate immune system, as it can effectively sense viral invasion and trigger strong inflammatory response [89]. Two signals are required for NLRP3 inflammasome activation [90]. The first signal, also called the priming signal, will activate NF-κB and promote the transcription of proinflammatory genes including NLRP3, pro-IL-1β, and pro-IL-18. The second signal, also known as the activation signal, will trigger the assembly of NLRP3 inflammasome [90]. 

**Figure 2:**
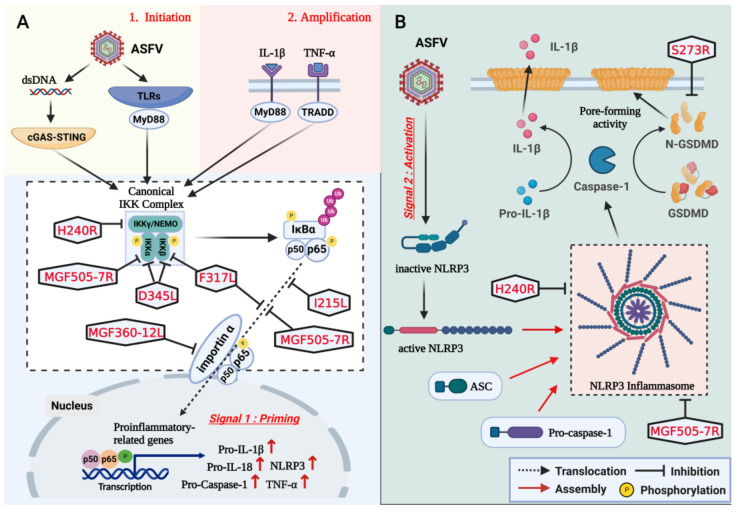
Schematic overview of inhibition of NLRP3 inflammasome activation and GSDMD-mediated pyroptosis by ASFV proteins in the latest reports. (**A**) ASFV pF317L, pH240R, pMGF505-7R, pD345L, pI215L, and pMGF360-12L inhibit the transcription of proinflammatory genes by targeting canonical NF-κB pathway. Mechanistically, pF317L inhibits the phosphorylation of IKKβ and the nuclear translocation of NF-κB, pH240R mediates the degradation of NEMO, pMGF505-7R and pI215L inhibit the nuclear translocation of NF-κB, pD345L directly interacts with IKKα and IKKβ, and pMGF360-12L competitively inhibits the interaction between p65 and importin-α. (**B**) ASFV pH240R and pMGF505-7R inhibit the assembly of inflammasome, and pS273R inhibits the GSDMD-mediated pyroptosis. Mechanistically, pH240R suppresses NLRP3 oligomerization, pMGF505-7R directly interacts with NLRP3, and S273R cleaves N-terminal of GSDMD.

To counteract, ASFV can encode multifunctional proteins to inhibit the activation of NF-κB, restrict the nuclear translocation of NF-κB, and disrupt the assembly of NLRP3 inflammasome by targeting the two signals (Figure 2). Firstly, one of the crucial steps in NF-κB activation is the phosphorylation of inhibitor of NF-κB alpha (IκBα) by canonical IκB kinases (i.e., IKKα and IKKβ) [91]. The canonical IκB kinases (IKKs) associate with an adapter protein NEMO (also known as IKKγ) to form “the canonical IKK complex” through their NEMO-binding domain [92]. NEMO without kinase activity usually acts as a ubiquitin-binding protein whose interaction with polyubiquitin chains is imperative for the canonical IKKs activation [93,94,95]. Nevertheless, ASFV-encoded capsid protein pH240R can mediate proteasome- and lysosome-dependent degradation of NEMO to ultimately block the activation of NF-κB [96]. As reported, phosphorylation of serine residues at positions 176 and 180 in IKKα or at positions 177 and 181 in IKKβ can make their kinase activity activated [97]. ASFV-encoded pF317L can inhibit the phosphorylation of IKKβ and further suppress NF-κB activation by decreasing phosphorylation and degradation of IκBα [98]. Additionally, ASFV pD345L and pMGF505-7R have both proven to interfere with NF-κB activation through their interaction with IKKα and/or IKKβ, but the mechanisms have not been deeply explored [99,100]. 

Secondly, NF-κB is normally associated with IκBα to form an inactive complex and sequestered in the cytoplasm [101]. In response to viral infection, NF-κB can be activated and translocate from cytoplasm into nucleus to exert its function on the transcription of proinflammatory genes [101]. However, ASFV-encoded pMGF360-12L can interfere with the nuclear import of NF-κB by competitively inhibiting the interaction between p65 and importin-α or karyopherin-α (KPNA) subtypes [102]. Moreover, three multifunctional proteins, namely pI215L (UBCv1), pMGF505-7R (A528R), and pF317L, have all been identified to restrict the nuclear translocation of NF-κB by performing immunofluorescence assay, but the mechanisms are not fully clarified [103,104]. Thirdly, upon activation, the sensor protein NLRP3 will recruit apoptosis-associated speck-like protein containing a CARD (ASC) and pro-caspase-1 to form a multiprotein complex called NLRP3 inflammasome [105]. However, ASFV pH240R can disrupt inflammasome assembly by suppressing NLRP3 oligomerization [96]. In addition, pMGF505-7R can also bind to NLRP3 to disturb the formation of inflammasome, but the mechanism is not further elucidated [100].

Of note, the assembly of NLRP3 inflammasome will further promote the activation of pro-caspase-1 [106]. Activated caspase-1 can cleave gasdermin D (GSDMD) in the interdomain linker to release the N terminal fragment of GSDMD (GSDMD-NT) [107]. GSDMD-NT oligomerizes in the cytomembrane and forms pores, which induces the lytic form of death-termed pyroptosis [107]. Pyroptosis functions as one of host defense mechanisms to restrict viral replication and facilitate the elimination of virus-infected cells [108]. As reported, viruses can utilize viral proteases to regulate pyroptosis [109,110]. Recently, Zhao et al. revealed that the ASFV-encoded pS273R can inhibit pyroptosis through noncanonical cleavage of swine GSDMD [111]. In the late stage of ASFV infection, pS273R cleaves GSDMD at G107-A108 to produce a shorter GSDMD-NT (N1~107) [111]. Unlike the canonical GSDMD-NT (N1~279) produced by caspase-1, GSDMD-NT (N1~107) loses its pore-forming activity on cytomembrane and is unable to induce pyroptosis [111]. Therefore, it is reasonable to speculate that GSDMD-mediated pyroptosis may be radically inhibited during ASFV infection. 

## 4. Effects of Immune Evasion on Viral Replication and Virulence

Innate immunity serves as the crucial line of defense-exerting functions in protection against viral invasion. However, IFN-I activity and inflammatory response, the two principal components of innate immunity, are significantly blocked by ASFV proteins. The relevant mechanisms have already been elaborated above. It is reasonable to presume that antagonisms of ASFV proteins towards innate immunity may promote its own proliferation and alter biological characteristics of virus. Thus, the implications that ASFV proteins-mediated immune evasion pose on viral replication, pathogenicity, and virulence in vivo should be fully investigated, which may provide rational design of LAVs. 

Here, immunosuppression-related genes H240R, MGF505-7R, E184L, I226R, and A137R are highlighted, referring to the latest reports on ASF LAVs. After deleting these genes individually from highly virulent ASFV strains, a significant decrease in pathogenicity and virulence can be observed in the mutants (Table 2). Previous studies have confirmed that deletion of the H240R gene will enhance NLRP3-mediated inflammatory responses, resulting in the attenuation of ASFV [112]. Of note, MGF505-7R has proven to be a multifunctional protein, playing crucial roles in suppressing cGAS-STING-mediated IFN-I production, IFN-II-induced antiviral response, and NLRP3 inflammasome activation [65]. Indeed, deletion of the aMGF505-7R gene does make ASFV trigger higher level of IFN-I and IL-1β in pigs, which may contribute to its attenuation. Although ASF LAV candidates, which are constructed by deleting a single gene (e.g., E184L, I226R, A137R), also exhibit attenuated characteristics to different extent, their mechanisms still remain to be further explored. 

In addition, ASF LAV candidates SY18ΔI226R, Georgia/2010-ΔA137R, and Georgia/2010-ΔE184L all induce medium-to-high level of viremia (Table 2). The level of viremia reflects the level of viral replication in vivo [113]. Thus, these data demonstrate that single deletion of a gene (e.g., E184L, I226R, A137R) does not significantly affect ASFV replication in vivo. However, E184L, I226R, and A137R can all significantly inhibit viral replication in vitro through interfering with cGAS-STING-mediated IFN-I production. ASFV proteins may mediate antagonisms in vivo in a compensatory but not redundant manner. MGF360-9L and MGF505-7R, two inhibitors of IFN-induced antiviral response, exhibit synergistic restriction on viral replication in vivo. Indeed, combinational deletions of MGF360-9L and MGF505-7R attenuate ASFV and induce a much lower level of viremia than the parental strain does in vivo [114]. 

**Table 2:** Viremia, clinical signs, and death in LAVs-inoculated pigs compared with parental strains.

LAV Candidates vs. Parental Strains	Viremia (Replication)	Clinical Signs (Pathogenicity)	Death (Virulence)	Reference
HLJ/18	Unknown	Yes, 6/6	Yes, 6/6	[112]
HLJ/18-ΔH240R	Unknown	No, 0/6	No, 0/6	
HLJ/18	Yes, high	Unknown	Yes, 5/5	[100]
HLJ/18-Δ7R	Yes, medium	Unknown	Yes, 2/5	
Georgia/2010	Yes, high	Yes, 5/5	Yes, 5/5	[115]
Georgia/2010-ΔE184L	Yes, medium to high	Yes, 2/5	Yes, 1/5	
SY18	Yes, high	Yes, 5/5	Yes, 5/5	[116]
SY18-ΔI226R	Yes, medium to high	No, 0/5	No, 0/5	
Georgia/2010	Yes, high	Yes, 5/5	Yes, 5/5	[117]
Georgia/2010-ΔA137R	Yes, medium to high	No, 0/5	No, 0/5	
CN/GS/2018	Yes, high	Yes, 6/6	Yes, 6/6	[114]
CN/GS/2018-Δ9L/Δ7R	Yes, low	No, 0/6	No, 0/6	

## 5. Future Perspective

**Figure 3:**
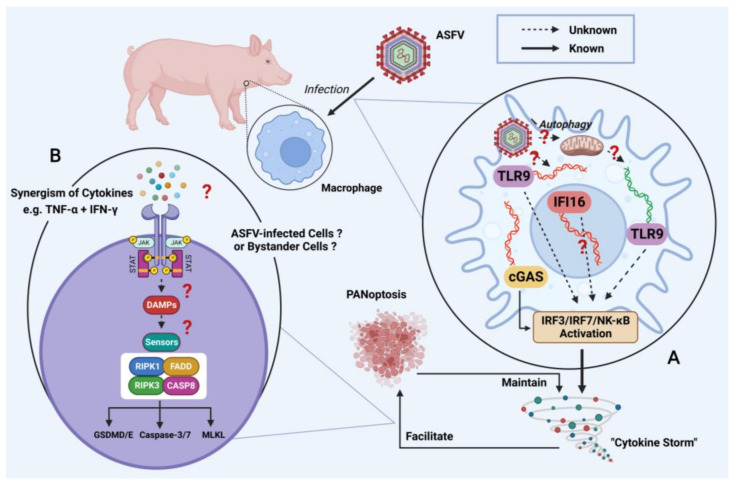
Schematic overview of scientific hypotheses proposed in this review. (**A**) Other nucleic acid sensors may be involved in ASFV detection. Mechanistically, TLR9 may directly recognize the unmethylated CpG DNA in the ASFV genome somehow or by indirectly sensing the ASFV-induced mitophagy-mediated mtDNA release. IFI16 may recognize ASFV through binding to viral DNA or sensing ASFV-induced host DNA damage. (**B**) Synergism of multiple cytokines may facilitate the PANoptosis; in turn, PANoptosis may sustain the ASFV-induced cytokine storm status.

The availability of safe and efficient vaccines is required for the control and eradication of ASF epidemics [118]. Thus, works contributing to the rational development of protective ASF vaccines should be a high priority [119]. The prerequisite for developing such effective vaccines is to better understand how ASFV antagonizes host immunity and pathogenesis of ASFV infection [120]. Unfortunately, there are still deep gaps that should be filled in above research fields (Figure 3).

Firstly, the involvement of nucleic acid sensors in ASFV detection has not been thoroughly understood. Although recent studies have highlighted that the cGAS/STING pathway plays predominant roles in resisting ASFV infection, other DNA sensors may be involved in ASFV recognition as well [27]. In particular, the potential functions of toll-like receptor 9 (TLR9) and interferon gamma inducible protein 16 (IFI16) have not yet been well investigated. TLR9 is a DNA-sensing receptor expressed in professional innate immune cells such as DCs and macrophages [121]. It recognizes unmethylated CpG-rich DNA of microbial origins [122]. TLR9 activation promotes the synthesis of proinflammatory cytokines such as IL-12, IL-6, and TNF-α, which is consistent with those induced by ASFV [123,124]. Moreover, recent studies have indicated that knockdown of TLR9 significantly down-regulated ASFV-Δ7R-triggered pro-IL-1β transcription [100]. These data suggest that TLR9 might be involved in the recognition of ASFV invasion. Most importantly, there is abundant unmethylated CpG DNA within the ASFV genome [125]. However, the ASFV genome remains wrapped by the thick core shell until it is released into the cytoplasm. Thus, it is not clear how unmethylated CpG DNA in ASFV genome can be exposed to the TLR9. To the best of our knowledge, endogenous mitochondrial DNA (mtDNA), whose CpG motifs are also unmethylated, is the ligand for TLR9 [126]. Therefore, special cases should be considered in which ASFV infection may indirectly activate the TLR9 pathway by triggering mitophagy-mediated mtDNA release [127]. To date, the relationship between the ASFV and TLR9 signaling pathway has not been elucidated yet. In addition, IFI16 can recognize many DNA viruses and detect genomic lesions following DNA damage [128]. Previous studies confirmed that the host DNA damage response (DDR) is activated from the early stage of ASFV infection [129]. Therefore, it is reasonable to speculate that IFI16 may recognize ASFV through binding to viral DNA or sensing ASFV-induced host DNA damage. More nucleic acid sensors involved in ASFV recognition need to be identified in the near future. 

Secondly, the pathogenesis of ASFV has not been clearly elucidated. Hyperactivation of the immune system will result in a sharp and robust increase of proinflammatory cytokines, leading to a “cytokine storm” [130]. Cytokine storm syndrome, defined as a collection of severe clinical manifestations, is characterized by systemic inflammation, multi-organ failure, etc. [130]. As previously reported, the viruses (e.g., SARS-CoV-2, pseudorabies virus)-induced cytokine storm seems to be associated with their highly pathogenic infections, which could even give rise to rapid fatalities [131,132]. Indeed, ASF is also a devastating disease with high mortality in pigs. Recently, Wang et al. confirmed that the cytokine storm is involved in the pathogenesis of ASFV [124]. Upon the infection of type II virulent ASFV SY18 in domestic pigs, they characterized the kinetics of representative cytokines (e.g., interferons, interleukins, growth factors, tumor necrosis factors, and chemokines) circulating in vivo. As a result, they observed ASFV-induced cytokine storm in vivo. ASFV-infected pigs showed severe clinical symptoms from 3 days post inoculation (dpi) and died from 7 to 8 dpi. Of interest, except for IFN-γ, the majority of proinflammatory cytokines had a robust and sustained elevation throughout the ASFV SY18 infection. In addition, a two-time increase of the levels of TNF-α, IL-1β, IL-6, and IL-8 presented an irreversible immune status as well. Notably, Kanneganti et al. found that synergism of TNF-α and IFN-γ induced PANoptosis, and in turn, TNF-α and IFN-γ-mediated PANoptosis maintained the status of “cytokine storm”, which is extremely critical for the pathogenic processes of COVID-19 [133]. PANoptosis is activated by specific stimulus and modulated by its core “PANoptosome” complex that provides a molecular scaffold for extensive crosstalk of key molecules among pyroptosis, apoptosis, and necroptosis [134]. Whether PANoptosis is involved in the maintenance of ASFV-induced “cytokine storm” status and which cytokines synergize to drive the PANoptosis during ASFV infection deserve further investigation.

## Data Availability

All data generated or analyzed during this study are included in this published article.
